# The Distribution of Autoantibodies by Age Group in Children with Type 1 Diabetes versus Type 2 Diabetes in Southern Vietnam

**DOI:** 10.3390/jcm12041420

**Published:** 2023-02-10

**Authors:** Quynh Thi Vu Huynh, Minh Thi Tuyet Trinh, Khang Kim Doan, Ban Tran Ho, Szu-Chuan Shen, Tung Huu Trinh, Thanh Hoa Vo, Nguyen Quoc Khanh Le, Ngan Thi Kim Nguyen

**Affiliations:** 1 Department of Pediatrics, Faculty of Medicine, University of Medicine and Pharmacy at Ho Chi Minh City, Ho Chi Minh City 700000, Vietnam; 2 Department of Nephrology and Endocrinology, Children’s Hospital 2, Ho Chi Minh City 700000, Vietnam; 3 Faculty of Medicine, University of Medicine and Pharmacy at Ho Chi Minh City, Ho Chi Minh City 700000, Vietnam; 4 Le Van Thinh Hospital, Ho Chi Minh City 700000, Vietnam; 5 Department of Pediatric Surgery, Faculty of Medicine, University of Medicine and Pharmacy at Ho Chi Minh City, Ho Chi Minh City 700000, Vietnam; 6 Children’s Hospital 2, Ho Chi Minh City 700000, Vietnam; 7 Programs of Nutrition Science, School of Life Science, National Taiwan Normal University, Taipei 11677, Taiwan; 8 Department of Science, Pharmaceutical and Molecular Biotechnology Research Center, South East Technological University, X91 K0EK Waterford, Ireland; 9 Professional Master Program in Artificial Intelligence in Medicine, College of Medicine, Taipei Medical University, Taipei 106, Taiwan; 10 Research Center for Artificial Intelligence in Medicine, Taipei Medical University, Taipei 106, Taiwan; 11 Translational Imaging Research Center, Taipei Medical University Hospital, Taipei 110, Taiwan

**Keywords:** islet cell autoantibodies (ICAs), glutamic acid decarboxylase 65 autoantibodies (GADAs), pediatric diabetes, children obesity

## Abstract

Asian children are increasingly being diagnosed with type 1 diabetes (T1D) or type 2 diabetes (T2D), and the presence of coexisting islet autoimmune antibodies complicate diagnosis. Here, we aimed to determine the prevalence of islet cell autoantibodies (ICAs) and glutamic acid decarboxylase 65 autoantibodies (GADAs) in children with T1D versus T2D living in Vietnam. This cross-sectional study included 145 pediatric patients aged 10.3 ± 3.6 years, with 53.1% and 46.9% having T1D and T2D, respectively. ICAs were reported in only 3.9% of pediatric T1Ds, which was not significantly different from the 1.5% of those with T2D. Older children with T1D were positive for either ICAs, or ICAs and GADAs (5–9 and 10–15 years), whereas only a small proportion of children aged 0–4 years were positive for GADAs (18%). Notably, 27.9% of children with T2D aged 10–15 were positive for GADAs, and all were classified as overweight (*n* = 9) or obese (*n* = 10). GADAs were more commonly observed in T1D patients younger than four years than ICAs, which were more prevalent in older children (5–15 years). Even though few children with T2D carried ICAs and GADAs, finding a better biomarker or an appropriate time to confirm diabetes type may require further investigation.

## 1. Introduction

The problem of diabetes in children poses significant public health implications due to the serious complications it can cause in later life as a result of either chronic insufficient insulin secretion or impaired insulin action in later years [1]. According to the latest ADA 2021 classification, diabetes in children is classified into type 1 diabetes (T1D)—with a known mechanism underlying the appearance of islet cell autoantibodies—and type 2 diabetes (T2D), characterized by a progressive loss of adequate β-cell insulin secretion, frequently with a background of insulin resistance [1]. Worldwide, the incidence and prevalence of T1D and T2D among children is increasing—especially among the Asian population [2,3,4]. The early onset of diabetes means a more prolonged lifetime exposure to hyperglycemia, which is highly associated with adverse outcomes in adulthood [5]. Remarkably, children with T2D are prone to develop earlier and more severe microvascular and cardiovascular disease than those with T1D [6,7,8]. Therefore, it is essential to classify individuals with T1D or T2D at the time of diagnosis to determine appropriate and effective therapies [9].

As mentioned, T1D pathogenesis has been linked to a state of β-cell destruction in the presence of autoantibodies, which are considered an important factor in distinguishing the various types of diabetes (autoimmune—T1A or idiopathic—T1B, or T2D). Among the well-known T1D-linked autoimmune markers, islet cell autoantibodies (ICAs) and glutamic acid decarboxylase 65 autoantibodies (GADAs) are widely used [10,11]. Importantly, the factors that trigger the autoimmune phenomenon in children with a genetic susceptibility to T1D remain unknown. Evidence linking the lower ICA frequency of black patients compared to their white counterparts [12] or Caucasians [13] is partially explained by ethnicity and genetics, along with their environmental interactions. Interestingly, the presence of autoimmune markers among Asian children is lower than in non-Asians [14], as there are still several T1B subtypes characterized by the absence of insulitis and diabetes-related antibodies [15].

In Vietnam, a previous publication revealed that a high number of T1D cases presenting with diabetic ketoacidosis in young adults are negative for pancreatic ICAs. Additionally, although the authors reported a low prevalence of T1D and T2D among children aged 11–14 years (1.04), there has been a lack of prevalence rates published for T1D and T2D in younger children and descriptions of autoimmune markers [16]. The lower frequency of ICAs in children with T1D onset before five years of age may be due to a more rapid disappearance of islet cell antigens than in patients with a later onset [13]. Due to there being little information on autoantibodies in this racial group so far to our knowledge, this study aimed to investigate the existence of autoantibodies in T1D and T2D among younger Vietnamese children aged 1–15. We also examined the prevalence of positive autoimmune makers stratified by age group. Based on the findings of this study, we will be able to gain a deeper understanding of the epidemiology of diabetes in Vietnam and the etiology of diabetes autoantibodies in Asian populations.

## 2. Materials and Methods

### 2.1. Study Population

This study was a retrospective cross-sectional investigation describing cases from medical records gathered at Children’s Hospital 2 over five years (2015–2020). The inclusion criteria for the 145 pediatric patients were as follows: (a) Patients aged from 6 months to 15 years; (b) Were diagnosed as having diabetes mellitus for the first time upon being admitted to the hospital, using the 2019 Classification and Diagnosis of Diabetes, with either HbA1c > 6.5%, fasting blood glucose > 126 mg/dL, or random blood glucose >200 mg/dL. Thereafter, patients were divided into two groups: (1) T1D, referred to as children whose diabetic signs and symptoms occurred early in life, had no family history of diabetes, were not overweight or obese, and had a low fasting C-peptide (<1.1 ng/mL), and (2) T2D patients, who developed at pubertal age, were overweight or obese, and had high C-peptide levels (>1.1 ng/mL) or high fasting insulin (>2.6 mcg/mL). After diagnosis and treatment, we repeatedly assessed serum C-peptide levels one month later to ensure that glucose toxicity would not lead to an underestimation of C-peptide levels. The T1D patients were prescribed insulin. In contrast, T2D patients were given Metformin (if HbA1C < 8.5%), or insulin (if HbA1C ≥ 8.5%) in the short term (2–6 weeks) before transitioning to Metformin. In addition, we checked responses to treatment after six months to confirm the diagnosis.

**Figure 1:**
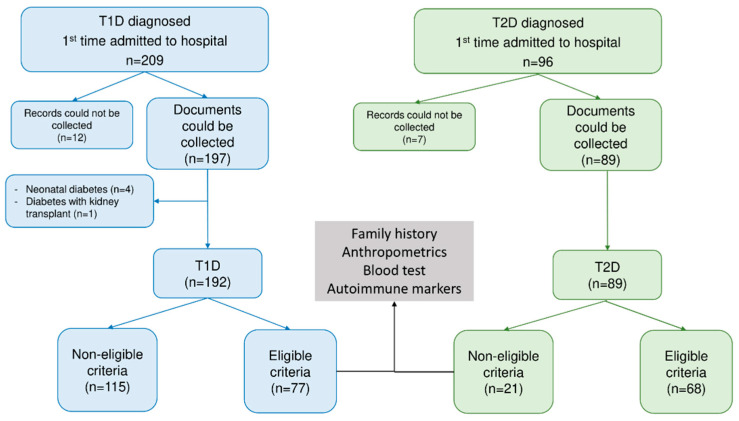
Flowchart for the study. Abbreviation: T1D—type 1 diabetes, T2D—type 2 diabetes.

All recruited patients were measured for pancreatic islet autoantibodies, such as ICA and GADA and thyroid autoantibodies. The study excluded any participants meeting any of the following criteria: (c) Medical records could not be collected; (d) Neonatal diabetes or monogenic diabetes; (e) Post-transplant diabetes mellitus; (f) Non-T1D and non-T2D. Figure 1 illustrates the flowchart for the study recruitment (Figure 1).

### 2.2. Data Collection

We collected demographic and clinical information, including age at diagnosis, gender, diabetic family history, insulin usage, diabetes-related symptoms, polydipsia, polyuria, polyphagia, unintentional weight loss, diabetes ketoacidosis (DKA), and acanthosis nigricans from parents, caregivers, and hospital personnel. In order to measure body weight and height, participants were instructed to wear light clothing and be barefoot, and then we calculated their body mass index (BMI) using the following formula: BMI = (Bodyweight)/(height^2^) in kilograms per square meter. Obesity was defined, based on the ages of the children, according to the median WHO Child Growth Standards median [17]. Children under five years of age were defined as overweight when weight-for-length/height or BMI-for-age > 2 standard deviations (SD) and ≤ 3 SD and as obese when weight-for-length/height or BMI-for-age > 3 SD. Children aged 5–19 years were determined as overweight when BMI-for-age > 1 SD and obese when BMI-for-age > 2 SD [17].

A serum sample from a patient was also used to assess the antibody assay methodology for islet cell autoantibodies (ICAs), glutamic acid decarboxylase antibodies (GADAs), thyroperoxidase antibodies (anti-TPOs), thyroglobulin antibodies (anti-TGs), and thyrotrophin receptor antibodies (TRAbs), which were stored in aliquots at −75 °C until the analysis was performed. Autoantibodies for pancreatic islet-cells consisting of ICAs and GADAs were detected using a qualitative enzyme-linked immunosorbent assay (ELISA; ImmunomatTM, DGR Instruments GmbH, Germany). Samples with ratio values of 0.95 U/mL or less showed a low level of ICAs (negative result), and samples between 0.95 and 1.05 U/mL were considered indeterminate (borderline). Positive results (high levels of ICAs) were determined by ratio values greater than 1.05 U/mL. Regarding GADA detection levels, a GADA ratio of less than 1 U/mL indicates a low antibody level (negative result), while an antibody ratio of 1 to 1.05 demonstrates a borderline level. Ratio values greater than 1.05 U/mL indicate a positive result for GADAs.

Autoantibodies for the thyroid: With a quantitative electrochemiluminescence immunoassay (ECLIA), thyroid antibodies were detected (Roche Cobas e602). For TRAb, TPOAb, and TGAb, a positive result was any value over 1.22 IU/L, 34 IU/mL, and 115 IU/mL, respectively.

### 2.3. Statistical Analysis

Data were presented as percentages for the categorical variables and mean ± SD for the continuous variables. To estimate the 95% confidence intervals (CIs) of T1D and T2D prevalence rates, the 25th value of the ranked difference, as well as the 95th value of the ranked difference, can be used for 1000 bootstrap resamples of the mean difference. Pearson’s chi-squared test was used to determine the differences between categorical variables among T1D and T2D subjects. On the other hand, the independent Student’s *t*-test was used to compare the means of continuous data between T1D and T2D subjects. Statistics could be considered significant if there was a *p*-value below 0.05. As part of the statistical analysis, we used R Statistics software (version 3.6.3, R core team, 2020) to perform the analysis.

## 3. Results

### 3.1. Demographic Characteristics of the Study Population with T1D and T2D

Of 145 diabetic patients aged 10.30 ± 3.64 years (ranging from 1–15 years) and with a BMI of 19.45 ± 10.62 kg/m^2^, T1D and T2D accounted for 53.1% (95% CI 44.8–60.7%) and 46.9% (95% CI 39.3–55.2%), respectively. The study population was dominated by female subjects (female:male ratio = 1.4:1.0); however, no statistical differences in gender were noticed. A significant difference was observed in the distribution of the age groups between children with T1D and those with T2D (*p* < 0.001). In further detail, there was a high prevalence of T1D among young children—particularly those under the age of nine years (65.9%). By contrast, most of the subjects diagnosed with T2D were older children, with an age range between 10 and 15 years old (91.2%). There were significant differences in the family history of diabetes between children with T1D and T2D. Notably, the highest rates belonged to those with second-degree relatives with diabetes in both T1D and T2D children (Table 1).

### 3.2. Clinical Characteristics of the Study Population with T1D and T2D

Typically, children who have been diagnosed with T1D or T2D present with polyuria and unintended weight loss, which have been found to be significantly more prevalent in the T1D population. In addition, children with T1D appeared to have a significantly higher rate of insulin use, DKA, and urine ketone incidences than those with T2D at admission time (26% vs. 0%, 26% vs. 0%, and 93.5% vs. 27.9%, respectively) in this study, whereas subjects with T2D presented with significantly higher levels of obesity and being overweight, as well as signs of insulin resistance (acanthosis nigricans) when compared to those with T1D (98.5% vs. 3.9% and 48.5% vs. 0%, respectively; Table 2).

In terms of the blood test, it was found that T1D patients had significantly higher concentrations of blood glucose compared to those who had T2D (419.69 ± 167.50 vs. 280.35 ± 125.47 mg/dL, *p* < 0.001). T1D patients, on the other hand, had significantly lower levels of fasting insulin (2.90 ± 1.63 vs. 28.73 ± 27.25 µU/mL, *p* < 0.001) and C-peptide levels (0.42 ± 0.27 vs. 3.17 ± 2.16 ng/mL, *p* < 0.001) compared to those with T2D. No significant difference in HbA1c values between children with T1D and T2D was observed (Table 2).

### 3.3. Prevalence of Autoantibodies among Children with T1D and T2D

**Figure 2:**
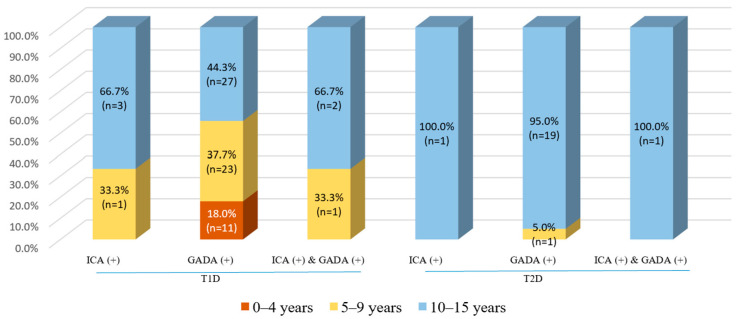
The positive rate of ICA and GADA tests, stratified by age groups, among children with T1D and T2D. Abbreviation: ICA—islet cell autoantibody, GADA—glutamic acid decarboxylase 65 autoantibody, T1D—type 1 diabetes, T2D—type 2 diabetes.

There was a notable finding in this study in that only 3.9% (*n* = 3) of 77 patients with T1D had positive ICAs, while 96.1% (*n* = 74) had negative ICA tests. At the same time, 79.2% of patients with T1D presented with positive GADA tests—significantly higher than those with T2D (79.2% vs. 29.4%, *p* < 0.001). Additionally, we observed that patients with T2D had a limited number of positive ICA tests (1.5%). In terms of the combination of ICA and GADA status, we found that patients with T1D had a significantly higher rate of positive ICA and positive GADA tests than those with T2D (3.9% vs. 1.5%, *p* < 0.001). Similarly, concerning negative ICA tests, patients with T1D had a higher proportion of positive GADA tests than those with T2D. Intriguingly, none of the patients was observed to have positive ICA tests and concomitant negative GADA tests—even in T1D patients. As the age groups were significantly different between the T1D and T2D groups (Table 1), we presented the distribution of positive ICA and GADA tests in different age groups—illustrated in Figure 2. Regarding patients with T1D, older ones were positive to either ICAs or ICAs and GADAs (5–9 and 10–15 years), while only a small number of patients aged 0–4 years appeared to be positive for GADAs (18%). By contrast, a limited number of T2D patients with an age of 10–15 years were positive for either ICAs or GADAs. A notable finding was that of the 68 recruited T2D participants, 27.9% of children aged 10–15 years had a positive GADA test (19 out of 68), and all were classified as overweight (*n* = 9) or obese (*n* = 10; Figure 2).

The present study revealed that several patients with T2D were positive for TRAb (*n* = 4) and anti-TPO (*n* = 1). However, only two patients with T1D presented as positive for anti-TG, but no incidence was found in patients with T2D (Table 3).

## 4. Discussion

The present study examined the prevalence and distribution across ages of autoimmune antibodies among children with T1D and T2D living in southern Vietnam. Positive ICA tests were found in 3.9% of T1D patients and 1.5% of T2D patients; on the other hand, positive GADA tests were found in 79.2% of T1D patients and 29.4% of T2D patients. A limited number of patients had both antibodies (ICA-positive and GADA-positive) together, as identified in 3.9% of T1D patients and 1.5% of T2D patients. Only 18.0% of T1D patients aged 0–4 years had positive GADA tests (*n* = 11), and none had positive ICA tests. In T1D patients aged 5 to 9 years, 33.3% had positive ICA tests, 37.3% had positive GADA tests, and 33.3% had both, respectively. In addition, many more older patients with T1D (aged 10 to 15) were positive for autoimmune markers, including ICAs (66.7%), GADAs (44.3%), and both (66.7%). As opposed to this, patients with T2D who had positive ICA and GADA tests were aged 10 to 15, and none were aged 0–4.

Typically, T1D is classified as autoimmune (T1A) or idiopathic (T1B) diabetes. The former type is more common (80–90%) and is caused by the autoimmune destruction of the insulin-producing β-cells in the pancreas, resulting in insulin deficiency [18]. Meanwhile, the latter type is known as islet antibody–negative diabetes, with a profound loss of insulin secretion, and is reported mainly in Asia [14,18]. Similarly, we observed that 96.1% of patients with T1D were negative for ICAs, and so were potentially misclassified as having T1D based on clinical characteristics. In this case, GADAs—markers of the autoimmune nature of T1D persisting over many years—could be recruited. Despite having negative ICA tests, 75.3% of T1D patients had positive GADA tests, classifying these cases as autoimmune [19]. In contrast, it was found that 20.8% of patients with T1D were classified as having T1B as a result of negative ICA and GADA tests. In line with our findings, Libman and colleagues [12] reported that a total of 12% of black patients with T1D did not possess any islet antibodies (ICAs, GADA, and ICA512), compared with 4% of white patients. This difference in the autoantibody prevalence between pediatric black and white patients with T1D might be explained by differences in the onset and/or progression of insulin-dependent diabetes mellitus [12]. According to many works in the literature, there is a wide variation in the prevalence of positive GADA status among different ethnic groups, such as 79% in Germany [20] and Belgium [21], 73% in Taiwan, and 44.3% in Singapore [22]. Increasing levels of GADAs indicate an ongoing immune attack against pancreatic β-cells; however, this autoimmune marker requires a persistent elevation for at least six months following diagnosis [19], which is longer than the period we observed. Therefore, it was essential to follow up and re-examine these individuals carefully. Importantly, we could not find any statistical differences between children who had at least one positive ICA or GADA test and those who presented as all-negative with respect to blood glucose, hemoglobin A1c, insulin, and C-peptide levels at diagnosis—even in the presence of diabetes ketoacidosis (Supplementary Table S1). In contrast to our study, a previous study reported that 60% of T1D patients who were negative for ICAs and GADAs among young adults in Vietnam presented with ketoacidosis without clear evidence of humoral or autoimmune mediators [23]. It should be noted that similar findings have been reported in some other populations from Asia, suggesting that the findings may be generalizable across ethnic groups [24,25,26]. Therefore, further research is necessary to determine the role of the age of onset and the clinical manifestations of T1D in children and adults. Additionally, although other autoimmune diseases related to the thyroid gland have been documented as being associated with T1D [27], only two patients with T1D (2.6%) demonstrated positive thyroglobulin antibody (TGAb), but were negative for thyrotrophin receptor antibodies (TRAb) and TPO antibodies. These were older children who, aged 9–13 years, presented as negative for ICAs, but positive for GADAs. As stated, autoimmune thyroid disease is the most common disorder related to T1D, but its incidence varies significantly in different populations [11,25,26]; therefore, the current population may not be representative—this requires further mechanistic studies. Last but not least, the present study found that patients with T1D were significantly more likely to suffer ketoacidosis than those with T2D, which is a classic approach to ascertaining diabetes type [9].

There is considerable difficulty in distinguishing between diabetes types in children due to the overlap of clinical features such as polyuria, polydipsia, polyphagia, and even ketoacidosis [9]—this is consistent with the results of the current study, except for ketoacidosis. Based on insulin and C-peptide levels, obesity, acanthosis nigricans, and family history of T2D, higher frequencies were found in the T2D group than in the T1D group in the present study. Moreover, the study identified that 30.9% of children and adolescents with T2D with at least one autoimmune antibody against β-cells were possibly diagnosed with double diabetes, whose prevalence rate was consistent with previous studies [11,27,28]. The co-existence of T1D and T2D may increase complications and worsen outcomes, such as microvascular and metabolic disorders associated with T1D and macrovascular disorders associated with T2D [28,29,30]. Apart from the evidence of 27.3% of T2D adult patients having thyroid diseases, derived from a large cohort study, the incidence of thyroid disorders in children with T2D is not fully understood [31]. Notably, the current study showed that five patients with T2D carried thyroid autoantibodies (TRAb and TPO-Ab). Only one of these patients, aged 14 years, presented as positive for GADAs and ICAs, which may indicate hybrid diabetes or poor management of T2D hyperglycemia [29]. Although much more evidence has reported the co-existence of an underlying complex linkage between T2D and thyroid dysfunction [30,32], there has not been clear guidance on how frequently thyroid function should be monitored in patients with T2D—especially in children. Due to these factors, further research is necessary to determine the adverse complications associated with double diabetes and thyroid disease co-existing in children and adolescents, which needs to be addressed to develop an optimal glycemic control treatment regimen.

Though this is the first study investigating the existence of antibodies against β-cells in children with T1D and T2D in Vietnam, the present report still has some limitations. Firstly, we had to confront the limitations inherent in a cross-sectional design in obtaining data when patients were admitted to the hospital. Thus, the follow-up plan would be to undertake further monitoring and treatment strategies. Secondly, although ICAs and GADAs are commonly used in clinical studies, it would be more effective to examine other autoimmune markers—including insulinoma-associated antigen-2 (IA-2A), insulin autoantibodies (IAA), and autoantibodies Zinc transporter 8 autoantibodies (ZnT8A)—which could be used to predict early autoimmune T1D and to determine the type of diabetes [32]. Finally, the small sample size of the current study is not representative of the entire population; hence, further research should be conducted on children with T1D and T2D throughout the country in different regions.

## 5. Conclusions

In conclusion, the presence of autoimmune antibodies related to diabetes plays an important role in distinguishing diabetes types, which was of great interest in the present study. However, the prevalence of two common autoimmune markers—ICAs and GADAs—in children with T1D was not as high as expected, especially for the presence of ICAs. Pediatric patients with T1D had a low prevalence of thyroid autoimmune antibodies, contrary to the concept of the co-existence of autoimmune diseases. According to the age group, T1D patients younger than four years were more likely to have GADAs than ICAs—which were more commonly observed in older children (5–15 years). As far as T2D is concerned, the present study also found that a significant number of patients had ICAs and GADAs. However, only a small number of them were positive for thyroid-related autoantibodies. Supposedly, the evaluation of antibodies against β-cells and the thyroid gland in children who have suspected T1D and T2D should be noted at diagnosis and in the longer term, to better ascertain diabetes type and to identify the appropriate therapy to facilitate individualized care and management.

## Figures and Tables

**Table 1:** Demographic characteristics of pediatric patients with T1D and T2D.

Demographic Characteristics	T1D (*n* = 77)	T2D (*n* = 68)	*p*-Value *
Age in years at admission (mean ± SD)	8.60 ± 3.87	12.22 ± 2.11	**<0.001**
0–4 years	14 (18.2)	0 (0)	**<0.001**
5–9 years	29 (37.7)	6 (8.8)	
10–15 years	34 (44.2)	62 (91.2)	
Gender, *n* (%)			
Females	42 (54.5)	43 (63.2)	0.289
Males	35 (45.5)	25 (36.8)	
Family history of diabetes, yes (*n*, %)	24 (31.2)	42 (61.8)	**<0.001**
In first-degree relative	4 (5.2)	16 (61.8)	**<0.001**
In second-degree relative	19 (24.6)	21 (31.9)	
In first- and second-degree relative	1 (1.3)	5 (7.4)	
Anthropometry (mean ± SD)			
Height (cm)	123.71 ± 21.81	147.44 ± 27.06	**<0.001**
Body weight (kg)	23.77 ± 9.14	60.30 ± 16.10	**<0.001**
BMI (kg/m^2^)	11.31 ± 4.34	28.68 ± 7.65	**<0.001**
BMI-for-age (Z-score)	−1.13 ± 1.64	2.14 ± 0.64	**<0.001**

* X^2^ for categorical variables and Student’s *t*-test for continuous variables. Statistical significance is indicated by bold text. Age in years at admission was also the age at the time of diagnosis. Abbreviation: BMI—body mass index, T1D—type 1 diabetes, T2D—type 2 diabetes, SD—standard deviation.

**Table 2:** Clinical characteristics of pediatric patients with T1D and T2D.

Clinical Characteristics (Mean ± SD)/*n* (%)	T1D (*n* = 77)	T2D (*n*= 68)	*p*-Value *
Symptoms			
Polydipsia, yes	62 (80.5)	46 (67.6)	0.076
Polyuria, yes	64 (83.1)	45 (66.2)	**0.018**
Polyphagia, yes	30 (39.0)	36 (52.9)	0.092
Unintended weight loss	62 (80.5)	45 (66.2)	**0.050**
Overweight & obesity, yes (*n*, %)	3 (3.9)	67 (98.5)	**<0.001**
Acanthosis nigricans, yes (*n*, %)	0 (0)	33 (48.5)	**<0.001**
On insulin at diagnosis, yes (*n*, %)	20 (26.0)	0 (0)	**<0.001**
DKA at diagnosis	20 (26.0)	0 (0)	**<0.001**
Blood glucose at diagnosis (mg/dL)	419.69 ± 167.50	280.35 ± 125.47	**<0.001**
Glycosuria, yes (*n*, %)	72 (93.5)	57 (83.8)	0.063
Urine ketone, yes (*n*, %)	72 (93.5)	19 (27.9)	**<0.001**
Hemoglobin A1c (%)	12.36 ± 2.53	11.75 ± 2.24	0.128
Plasma insulin (µU/mL)	2.90 ± 1.63	28.73 ± 27.25	**<0.001**
C-peptide (ng/mL)	0.42 ± 0.27	3.17 ± 2.16	**<0.001**

* X^2^ for categorical variables and Student’s *t*-test for continuous variables. Statistical significance is indicated by bold text. Abbreviation: DKA—diabetes ketoacidosis, SD—standard deviation, T1D—type 1 diabetes, T2D—type 2 diabetes.

**Table 3:** Positive rate of autoantibodies of pediatric patients with T1D and T2D.

Autoimmune Markers	T1D (*n* = 77)		T2D (*n*= 68)		*p*-Value *
	*n*	%	*n*	%	
Positive ICAs	3	3.9	1	1.5	0.130
Positive GADAs	61	79.2	20	29.4	**<0.001**
Negative ICAs and negative GADAs	16	20.8	48	70.6	**<0.001** #
Positive ICAs and negative GADAs	0	0	0	0	
Negative ICAs and positive GADAs	58	75.3	19	27.9	
Positive ICAs and positive GADAs	3	3.9	1	1.5	
Positive TRAb	0	0	4	5.9	0.046 #
Positive TPO-Ab	0	0	1	1.5	0.469 #
Positive TG-Ab	2	2.6	0	0	0.498 #

* X^2^ for categorical variables. # Fisher’s exact test for categorical variables. Statistical significance is indicated by bold text. Abbreviation: ICAs—islet cell antibodies, GADAs—glutamic acid decarboxylase antibodies, anti-TPO—thyroperoxidase antibody, anti-TG—Thyroglobulin antibody, TRAb—thyrotrophin receptor antibody.

## Data Availability

Data from this study will be available upon request.

## Supplementary Materials

The following supporting information can be downloaded at: https://www.mdpi.com/article/10.3390/jcm12041420/s1, Supplementary Table S1: Clinical characteristics of pediatric patients with T1D whose ICAs and GADAs are both negative and at least one positive.
