# Supplementary material for: The Distribution of Autoantibodies by Age Group in Children with Type 1 Diabetes versus Type 2 Diabetes in Southern Vietnam
Source: J Clin Med. 2023 Feb 10;12(4):1420. doi: 10.3390/jcm12041420 (PMC9961333; doi:10.3390/jcm12041420)
Supplement: Supplementary file 1 [file jcm-12-01420-s001.zip › jcm-2136275-supplementary.pdf]

Supplementary Table S1. Clinical characteristics of pediatric patients with T1D whose ICAs and GADAs are both negative and at least one positive.

**Clinical characteristics** (mean ± SD)/ *n* (%)	**(-) ICAs & (-) GADAs (*n* =16)**	**At least one positive Ab (*n* =61)**	***P*-value***
Ages (yrs)	8.13 ± 4.41	8.72 ± 3.76	0.587
Symptoms			
Polydipsia, yes	10 (62.5)	52 (85.2)	0.071[#]
Polyuria, yes	10 (62.5)	54 (88.5)	**0.023**[#]
Polyphagia, yes	5 (31.5)	25 (41.0)	0.572
Unintended weight loss	9 (56.2)	53 (86.9)	**0.011**[#]
Height (cm)	117.15 ± 25.71	125.43 ± 20.56	0.178
Body weight (kg)	23.45 ± 10.62	23.86 ± 8.80	0.873
BMI (kg/m^2)	11.15 ± 5.05	11.35 ± 4.18	0.873
BMI-for-age (Z-score)	-0.44 ± 1.83	-1.32 ± 1.55	0.056
On insulin at diagnosis, yes (n, %)	6 (37.5)	14 (23.0)	0.336[#]
DKA at diagnosis	7 (43.8)	13 (21.3)	0.107[#]
Blood glucose at diagnosis (mg/dL)	446.43 ± 174.68	412.68 ± 166.34	0.477
Glycosuria, yes (n, %)	15 (93.8)	57 (93.4)	1.000[#]
Urine ketone, yes (n, %)	15 (93.8)	57 (93.4)	1.000[#]
Hemoglobin A_{1c} (%)	11.77 ± 2.46	12.51 ± 2.54	0.308
Plasma insulin (μU/ml)	2.58 ± 0.89	2.99 ± 1.77	0.382
C-peptide (ng/ml)	0.41 ± 0.35	0.43 ± 0.26	0.784

X^2 for categorical variables and Student's t-test for continuous variables.

[#] *Fisher's exact test for categorical variables.*

Statistical significance is indicated by bold text.

Abbreviation: BMI body mass index, DKA diabetes ketoacidosis, ICAs, islet cell antibodies; GADAs, glutamic acid decarboxylase antibodies
